# Pneumocystis Jirovecii Pneumonia: The Potential of KEX1, MSG1, and MSG2 as Key Antigens in Cytokine Release Assays

**DOI:** 10.3390/diagnostics15070793

**Published:** 2025-03-21

**Authors:** F. A. Ottilie Neumann, Markus Müller, Gregor Mattert, Sven Liebig, Victor Herbst, Dorinja Zapf, Til R. Kiderlen, Christian Linke, Franziska Arp, P. Markus Deckert, Stefan Lüth, Sandra Schwarzlose-Schwarck, Werner Dammermann, Mark Reinwald

**Affiliations:** 1 Department of Hematology, Oncology and Palliative Care, and Center for Translational Medicine, University Hospital Brandenburg an der Havel, Brandenburg Medical School Theodor Fontane, 14770 Brandenburg an der Havel, Germanymarkus.deckert@mhb-fontane.de (P.M.D.);; 2 Faculty of Health Sciences Brandenburg, Brandenburg Medical School Theodor Fontane, 14770 Brandenburg an der Havel, Germanyw.dammermann@uk-brandenburg.de (W.D.); 3 Department of Infectiology, Academic Medical Teaching Hospital, St. Joseph Krankenhaus Berlin Tempelhof, 12101 Berlin, Germany; 4 Department of Hematology, Oncology and Cancer Immunology, Campus Benjamin Franklin, Charité-Universitätsmedizin Berlin, Corporate Member of Freie Universität Berlin and Humboldt-Universität zu Berlin, 12203 Berlin, Germany; 5 Euroimmun Medizinische Labordiagnostika AG, 23560 Lübeck, Germany; 6 Department of Hematology and Oncology, Vivantes Auguste-Viktoria-Klinikum, 12157 Berlin, Germany; 7 Department of Gastroenterology, Center for Translational Medicine, University Hospital Brandenburg, Brandenburg Medical School Theodor Fontane, 14770 Brandenburg an der Havel, Germany

**Keywords:** pneumocystis jirovecii pneumonia, Kexin 1, MSG1, MSG2, interleukin-2 release assay, interferon-γ release assay, IL-17A, IL-17F

## Abstract

**Background/Objectives:** *Pneumocystis jirovecii pneumonia* (PJP) is the most frequently diagnosed AIDS-defining illness in Europe, with especially high mortality in HIV-negative patients caused by delayed diagnosis and low awareness. This study aims to evaluate cytokine release assays (CRA) to facilitate a less invasive and resource-efficient PJP specific diagnostic test. We focus on the *P. jirovecii* antigens Kexin 1 (KEX1), MSG1, and MSG2, which were identified in prior studies as immunologically relevant. **Methods:** Whole blood samples from 50 participants—22 healthy individuals and 28 immunocompromised individuals, including 8 with proven PJP—were stimulated in vitro with full-length and partial KEX1, MSG1, MSG2, and a combination of all three antigens (PJ-MIX). Following 24 h incubation at 37 °C, cytokine levels of IL-2, IFN-γ, IL-17A, and IL-17F were measured. **Results:** Stimulation with full-length KEX1, MSG1, MSG2, and PJ-MIX antigens induced higher IL-2 concentrations in the healthy control group compared to the groups IL-2 baseline levels and to the group of proven PJP cases. Similarly, stimulation with full-length KEX1, MSG1, and PJ-MIX elevated IFN-γ levels in the healthy control group compared to baseline IFN-γ levels. **Conclusions:** Our findings highlight the potential of IL-2 and IFN-γ release following stimulation with *PJ* antigens, with PJ-MIX eliciting the strongest and most significant responses, suggesting a cumulative antigen effect. This pilot study establishes a foundation for a PJP-specific CRA, deepening our knowledge of T-cell immunity against PJP. Clinically, such a test could, among other applications, evaluate at-risk patients who should receive prophylaxis and may consequently reduce PJP-related morbidity and mortality.

## 1. Introduction

### 1.1. Pneumocystis Jirovecii: An Unusual Fungus

*Pneumocystis* is a phylum of ascomycetes that is ubiquitously found and predominantly colonizes the alveoli in mammalian lungs [1]. Initially labeled *Pneumocystis carinii*, three different organisms are now distinguished: *Pneumocystis carinii*, *Pneumocystis murina*, and *Pneumocystis jirovecii* (*PJ*), with only *PJ* known to colonize and infect humans [2]. *PJ* was initially classified as a protozoan due to its atypical metabolism and unusual cellular structure. *PJ* is resistant to the most common antifungals [3] but is interestingly sensitive to the antibacterial agent cotrimoxazole [4,5], due to its cholesterol-containing cell membrane structure [6]. In comparison to other fungi, the cell wall of *PJ* is composed almost exclusively of glycoproteins dominated by multiple variants of the major surface glycoproteins (MSG) [7]. Due to its high host specificity, dependency, and yet unexplored nutrient requirements, *PJ* has not yet been cultured in vitro, which means that little is known about this organism, resulting in a substantially limited understanding of this atypical fungus [8].

### 1.2. Pneumocystis Jirovecii Pneumonia (PJP) and Epidemiology

*PJ* colonizes immunocompetent individuals, with initial contact typically occurring within the first two years of life [9]. In immunosuppressed hosts, *PJ* can cause severe pneumonia (PJP). PJP is the most common AIDS-defining disease in Europe and a leading cause of fungal pneumonia among children in Africa and Asia [10,11]. However, estimating its true prevalence is challenging due to the vulnerability of the patient population, the nonspecific symptoms, and the difficulties in diagnosing PJP. In a four-year observation period, more than 4500 confirmed PJP diagnoses were made in Spain [12]. The continuous advancement of antiretroviral therapy for HIV has led to a decrease in PJP cases among HIV^+^ populations; however, the incidence has shifted towards HIV-negative, immunocompromised individuals [13,14]. The clinical relevance of PJP is highlighted by its high mortality rate, which varies depending on the source, study, country, and time period, ranging from 36% to 60%, particularly among HIV-negative patients [11,15,16]. This elevated mortality is believed to be partly due to the low awareness and delayed diagnosis of PJP in non-HIV-infected individuals [17] as opposed to HIV^+^ patients. Late diagnosis of PJP and the delayed initiation of appropriate therapy significantly worsen patient outcomes [13].

### 1.3. The Challenge of Diagnosing PJP

Apart from technical aspects, PJP poses a diagnostic challenge due to its nonspecific clinical presentation, but chest computed tomography (CT) can offer initial diagnostic clues by revealing characteristic infiltrate patterns associated with PJP [18]. A definitive diagnosis is only confirmed when direct evidence of the pathogen in a specific quantity is obtained. Successful pathogen identification is most likely when infected material is collected directly from the site of manifestation [19,20]. Consequently, bronchoalveolar lavage (BAL) is regarded as the traditional diagnostic standard when PJP is suspected. Currently, the most widely used method for detecting *PJ* in lavage fluid is PCR, which provides genetic evidence of the organism. However, the mere detection of *PJ* in BAL fluid does not confirm *PJ* infection, as the individual may be colonized and not infected. Distinction between infection and colonization is made based on the quantity and reproductive behavior of the pathogen, utilizing specific PCR copy cutoffs [21,22]. Given that many patients with PJP are critically ill, BAL, being an invasive procedure with associated risks [23], is not warranted for every patient and additionally presents challenges in resource-limited settings. Recent diagnostic methods, including Next-Generation Sequencing (NGS) and the detection of PJP specific antibodies and antigens in various biological materials, have not yet been able to be integrated into clinical practice [24,25,26]. Due to the challenges in diagnosing PJP, prophylaxis remains the key strategy for PJP prevention, but deciding on who should receive prophylaxis for PJP also remains clinically challenging. While PJP poses a life-threatening risk to immunocompromised patients, individual risk varies based on factors such as CD4^+^ counts, steroid use, and chemotherapy regimens [27]. Despite the availability of prophylactic options like cotrimoxazole [28,29], clinical implementation remains inconsistent, particularly in patients with normal CD4^+^ counts. To overcome these challenges, targeted methods are needed to identify patients who would benefit most from prophylaxis. Moreover, novel, reliable, and less invasive diagnostic approaches for PJP are critically needed.

### 1.4. Cytokine Release Assays (CRA)

Interferon-gamma (IFN-γ) release assays (IGRAs) are immunological tests designed to assess an individual’s immune response by quantifying the release of IFN-γ. Rather than directly detecting the presence of a pathogen, IGRAs reflect the host’s immune response to it. IGRA test systems are well established and are utilized in routine daily practice, especially in the field of tuberculosis [30,31,32,33,34]. IGRAs exploit the fact that memory T-cells secrete IFN-γ upon exposure to pathogen-specific antigens, indicating prior infection. The released IFN-γ is then quantified by enzyme-linked immunosorbent assay (ELISA). Consequently, clinical conclusions can be drawn regarding the infectious as well as immunological status of the individual in relation to the specifically tested pathogen [33,34]. The primary advantage of cytokine release assays (CRA), like IGRAs evaluating the host immune response to a pathogen, lies in their ability to detect the pathogen even when serological or PCR-based methods fail to yield reliable results. Other cytokines, such as IL-2, are also applicable in the context of antigen stimulation and CRA [31,35,36].

### 1.5. The Idea of a CRA for Diagnosing PJP

Previous studies [25,37,38,39] have highlighted the immunological significance of Kexin (KEX1)—a serine endoprotease of *PJ* [6]—and variants of *PJ* MSG [30]. In their study, Tomás et al. identified IgM antibodies directed against KEX1 as well as MSG epitopes, reporting significantly higher anti-Kexin IgM titers in individuals infected with PJP compared to a healthy control cohort [25,38,39]. Similarly, Djawe et al. found elevated IgM antibody levels against recombinant fragments of MSG [40]. These findings provide compelling evidence of B-cell immunity to *PJ*, reinforcing the need to further explore the T-cell immunity in this context. Datta et al. have suggested an IGRA for diagnosing fungal infection [41]. For PJP, a significant challenge in diagnosing is the distinction between infection and colonization. Therefore, our objective is to identify an indirect approach for diagnosing PJP, focusing specifically on T-cell immunity and the release of the cytokines: IFN-γ, IL-2, IL-17A, as well as IL-17F. This study aims to fundamentally establish and evaluate a CRA from peripheral blood as a far less invasive and resource-efficient diagnostic tool for PJP in clinical practice. It is applicable for implementation in resource-limited healthcare settings, e.g., in non-industrialized countries. Through this study, we address a clinical need and hope to enhance the timeliness and reliability of diagnoses, expedite treatment initiation, and ultimately reduce mortality associated with PJP. To achieve this goal, our research project evaluated KEX1, MSG1, and MSG2 as specific antigens of *PJ* in a CRA using whole blood samples from immunocompromised patients, proven PJP cases, and healthy controls in a prospective multi-center study.

## 2. Materials and Methods

### 2.1. Participant Selection

This study was approved by the institutional review board of Landesärztekammer Brandenburg (Project ID: S20 (a)/2016, 7. Amendment approved 7 November 2023), which oversees human research, and informed consent was obtained from all participants. All participants were informed of the study’s purpose, procedures, and potential risks, and their right to withdraw at any time was explained. A signed declaration of agreement was required before inclusion in the study. This study included a total of 50 participants, consisting of 22 immunocompetent individuals and 28 immunocompromised individuals. The healthy controls consisted of hospital staff and medical students with no history of immunosuppression, including no use of immunosuppressive drugs, and were likely exposed to *PJ* through their work in the healthcare setting. The immunocompromised cohort, comprised of patients from the infectious diseases and oncology departments of three German Hospitals, included 20 individuals with current infections unrelated to PJP and 8 individuals with confirmed PJP, diagnosed by clinical presentation and positive PCR results from bronchoalveolar lavage (BAL) samples. Six of the eight confirmed PJP cases were HIV^+^, classified as AIDS stage C3 according to CDC criteria, which includes the presence of an AIDS-defining disease (PJP) and a CD4^+^ cell count below 200 cells/μL. The specific gender and age information from all participants are summarized in Table 1. A maximum of 20 mL of whole blood in lithium heparin and EDTA tubes was collected from each participant via peripheral venipuncture, with samples processed for cell stimulation within 24 h.

### 2.2. Flow Cytometric Analysis

Flow cytometric analyses were performed on blood samples collected in EDTA tubes from 18 participants using a FACSCelesta™ flow cytometer (Becton Dickinson, Franklin Lakes, NJ, USA). The cells were labeled using the antibodies CD3-BUV395 (SK7), CD4-BV786 (SK3), CD8-PerCP-Cy5.5 (SK1), CD14-FITC (M5E2), CD19-BUV737 (SJ25C1), CD45-BV480 (HI30), CD56-PE (B159), CD16-BV650 (3G8), CD28-BV421 (CD28.2), and HLA-DR-BV650 (G46-6) (all from Becton Dickinson, Franklin Lakes, NJ, USA). Human BD Fc Block™ (Becton Dickinson, NJ, USA) was added to block Fc-binding regions. The mix was incubated for 15 min at room temperature protected from light. Erythrocytes were lysed using BD FACS™ Lysing Solution (Becton Dickinson, Franklin Lakes, NJ, USA). Antibody volumes were titrated individually for optimal staining. Gates were defined using fluorescence minus one controls to ensure accurate analysis. As an internal quality control measure, duplicate measurements were performed for each participant to minimize potential measurement errors.

### 2.3. Antigen and Cytokine Selection

For antigen stimulation, five antigens were selected; Staphylococcal enterotoxin B (SEB) was used as a positive control due to its super-antigenic properties, along with Concanavalin A (ConA), a lectin with T-cell mitogenic properties mostly used in commercial IGRA-Tests. The specific *PJ* antigens selected were KEX1, MSG1, and MSG2, which were chosen based on previous studies demonstrating B-cell reactivity against these antigens [25,38,39,40]. However, since KEX1, MSG1, and MSG2 have not yet been established as antigens for T-cell stimulation, the exact protein sequences were first determined using the GenBank database from NCBI, followed by the synthesis of partial and full-length KEX1 (Genbank entry #AAM97495), full-length MSG1 (Genbank entry #AAA21645), and partial and full-length MSG2 (Genbank entry #AAA21779). The immunologically relevant regions of these antigens were identified using bioinformatics software to ensure the inclusion of key antigenic portions. The CD4^+^ T-cell immunogenicity was predicted using the immunogenicity prediction algorithm available on the IEDB website, as described by Dhanda et al. in 2018 [42]. We then ordered the custom synthesis of the antigens based on these protein sequences from the manufacturer Peptides&Elephants GmbH, Hennigsdorf, Germany. In addition, prototypic stimulation tubes containing the full-length antigens of MSG 1, MSG 2, KEX1, and a mixture of all 3 antigens named “PJ-MIX” were produced by EUROIMMUN AG, Lübeck, Germany. For our study, we selected the final antigen concentrations in the blood based on prior knowledge of antigen stimulation in CRA. Typically, pathogen antigens are used at 10 µg/mL, while SEB is used at 1 µg/mL and ConA at 10 µg/mL [31,32,43,44]. However, to ensure a comprehensive analysis, we opted to titrate the partial *PJ* antigens KEX1 and MSG2 in this initial study to assess their potential for cell stimulation. We tested concentrations ranging from 100 to 0.1 µg/mL to determine whether antigen concentration correlates with cytokine release (Table 2). The cytokines IFN-γ, IL-2, IL-17A, and IL-17F were chosen for measurement as indicators of T-cell immunity. IFN-γ is a critical cytokine produced by T-cells, mainly by T-helper 1 cells (Th1) and T-helper 2 cells (Th2). Its release in response to antigenic stimulation is an indicator of T-cell activation and immune competence [45]. IL-2 is known to be essential for T-cell proliferation, playing a key role in the expansion of antigen-specific T-cells. IL-2 presence provides interesting insight into the general functionality and activity of T-cells after an antigen exposure [46]. IL-17A and IL-17F, produced by Th17-cells, are key cytokines in antifungal defense. Their measurement reflects inflammation and the immune response to fungal control [47,48]. Measuring these cytokines offers a comprehensive view of (T-cell) immunity elicited by the chosen *PJ* antigens.

### 2.4. In-Vitro Cell Stimulation and Cytokine Measurement

Cell stimulation was performed under a class II biosafety cabinet, following national guidelines and regulations concerning the potential infectiousness of the materials used. Protective clothing, including a gown and gloves, was worn throughout the procedure. At the start of the stimulation, SEB, partial KEX1, and partial MSG2 antigens were diluted or titrated with the addition of 0.9% sodium chloride (NaCl). The final antigen concentrations used are summarized in Table 2. The cell stimulation process involved up to 21 different conditions for each individual. For each sample, the respective antigen, a defined volume of whole blood, and a 1 mg/mL glucose solution (for some conditions) were combined in up to 21 microtubes. The precise list of stimulation conditions for each subject is detailed in Table 2. After the reagents were added and thoroughly resuspended, the microtubes were incubated vertically and tightly closed at 37 °C for 24 h.

After 24 h of incubation, the cell stimulation was terminated. The microtubes were removed from the incubator and centrifuged at 12,000 rcf for ten min. Following centrifugation, the separated plasma from each stimulation condition was aliquoted and transferred into separate microtubes. To preserve the plasma samples till cytokine analysis, a dilution of sodium azide (NaN3) was added in a 1:200 ratio, resulting in a final concentration of 0.045% NaN3. The tubes were then stored at −20 °C until cytokine analysis was performed. An enzyme-linked immunosorbent assay (ELISA) was used to quantify the concentrations of IFN-γ, IL-2, IL-17A (#430106, #431806, #433914, Biolegend/Revvity, London, UK), and IL-17F (#DY1335B, R&D Systems/biotechne, Wiesbaden, Germany), respectively. Specifically, a sandwich ELISA format was employed for each cytokine, utilizing two specific antibodies: a captured antibody and a corresponding detection antibody. The enzyme used for detection was the streptavidin-polyHRP80 (SDT reagents, Baesweiler, Germany). The ELISA procedure itself was performed as established. To ensure test validity, each sample was measured at least twice simultaneously. A key challenge encountered was determining the appropriate sample dilutions for accurate cytokine quantification. For some immunocompromised subjects, significant volumes of samples were required to obtain precise cytokine concentration measurements. Conversely, the dilutions used for healthy subject samples were often too concentrated, leading to inaccurate cytokine determinations. Based on the varying stimulation conditions, sample dilutions ranged from 1:5 to 1:250,000 to ensure reliable cytokine concentration measurements.

### 2.5. Data Analysis

Photometric measurements were taken using a Spark 10M multimodal microplate reader (Tecan), and data analysis was performed using Microsoft Excel. For statistical analysis and a graphical representation of results, GraphPad Prism software (version 5) was used. Since we assumed a non-normal data distribution, partly due to varying immune status within the study cohort and the relatively small sample size, we applied non-parametric tests. To determine statistical significance, the Wilcoxon rank-sum test (Mann–Whitney U test) was used for comparisons between two independent groups. The Kruskal–Wallis test, followed by Dunn’s multiple comparisons test, was used to determine the adjusted *p* values when comparing more than two groups. The significance level of *p* < 0.05 was considered statistically significant, with a 95% confidence interval. We reported the mean, median, and standard error of the mean (SEM) for an initial overview of central tendencies and precision of sample mean estimates. While these values may be less robust for non-normal data, combined with non-parametric tests, they provide a clear representation of the data and their dispersion. Decimal places were rounded appropriately.

## 3. Results

### 3.1. Leukocytes, T-Cells, and CD4^+^/CD8^+^ Ratio

First, cell counts of total leucocytes, total T-lymphocytes, or CD4^+^ Th-lymphocytes were compared between the PJP patients, on the one hand, and the control groups of healthy and immunocompromised individuals, on the other hand. Calculated CD4^+^/CD8^+^ ratios were determined. The leucocyte count was significantly higher in the immunocompromised control group compared to the group of proven PJP cases (*p* = 0.0214, Table 3); the CD4^+^ Th-lymphocyte count was significantly higher in the healthy control group compared to proven PJP cases (*p* = 0.0006, Table 3) as well as the total T-lymphocyte count (*p* = 0.0092, Table 3). The CD4^+^/CD8^+^ ratio was significantly higher in the healthy control group compared to the group of proven PJP cases (*p* = 0.0052, Table 3). Table 3 shows all measured cell counts on the day of cell stimulation.

### 3.2. IFN-γ Release After Stimulation

**Figure 1:**
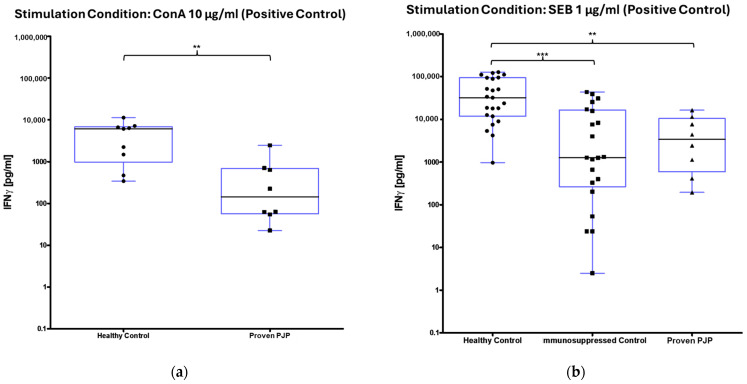
IFN-γ concentrations across all cohorts after stimulation with reference antigens (positive control) ^1^. (**a**) ConA stimulation significantly increased IFN-γ in healthy controls compared to proven PJP cases (** *p* = 0.0055) (Mann–Whitney U test); (**b**) SEB stimulation significantly increased IFN-γ in healthy controls compared to proven PJP cases (** *p* = 0.0078) and immunocompromised control group (*** *p* = 0.0002) (Kruskal–Wallis test with Dunn’s multiple comparisons). ^1^ Circles, squares and triangles represent the respective measurement of one participant from the cohort specified at the bottom of the figure.

**Figure 2:**
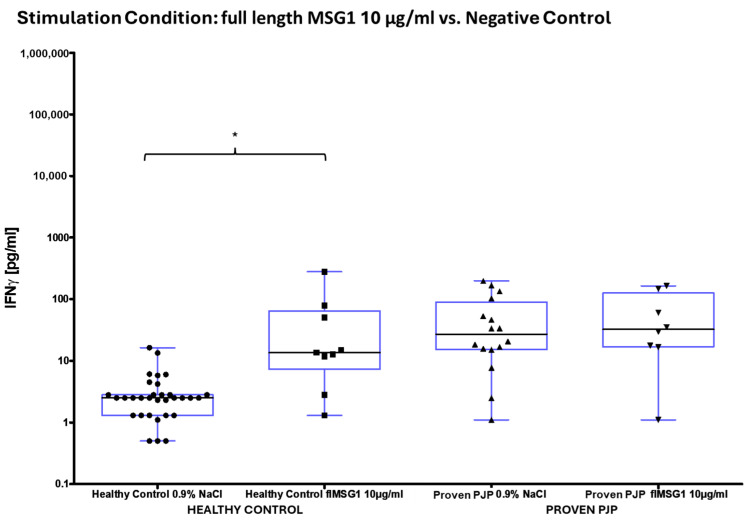
^1^ IFN-γ concentrations of healthy controls vs. proven PJP cases after full-length MSG1 stimulation compared to the baseline (NaCl). MSG1 significantly increased IFN-γ in healthy controls compared to the baseline (* *p* = 0.0188) (Kruskal–Wallis test with Dunn’s multiple comparisons).^1^ Circles, squares and triangles represent the respective measurement of one participant from the cohort and stimulation condition specified at the bottom of the figure.

**Figure 3:**
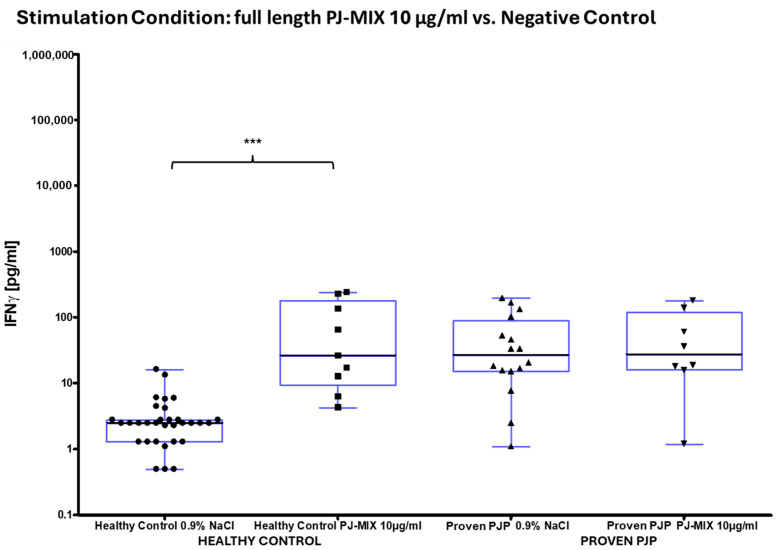
^1^ IFN-γ concentrations in healthy controls vs. proven PJP cases after full-length PJ-MIX stimulation compared to the baseline (NaCl). PJ-MIX significantly increased IFN-γ in healthy controls compared to the baseline (*** *p* = 0.0002) (Kruskal–Wallis test with Dunn’s multiple comparisons). ^1^ Circles, squares and triangles represent the respective measurement of one participant from the cohort and stimulation condition specified at the bottom of the figure.

Upon stimulation with ConA, a mitogen serving as the positive control, the measurable IFN-γ concentration was markedly elevated in the healthy control group compared to the group of proven PJP cases (*p* = 0.0055, Table 4, Figure 1a). Similarly, stimulation with SEB, a superantigen also utilized as a positive control, resulted in significantly higher IFN-γ concentrations in the healthy controls relative to the group of proven PJP cases (*p* = 0.0078, Table 4, Figure 1b) as well as compared to the immunocompromised control group (*p* = 0.0002, Table 4, Figure 1b). However, no significant difference was detected between the immunocompromised control group and the group of proven PJP cases following SEB stimulation. Baseline IFN-γ levels, defined as concentrations following 0.9% sodium chloride stimulation, were substantially higher in the proven PJP cases group compared to both the immunocompromised control group (*p* = 0.0012, Table 4) and the healthy control group (*p* < 0.0001, Table 4). However, there was no significant difference in baseline IFN-γ concentrations between the immunocompromised and healthy control groups. Stimulation with the full-length and partial MSG2, as well as with the partial KEX1 antigens, did not induce any significant difference in IFN-γ release among the cohorts. Furthermore, within each cohort, no significant changes in IFN-γ concentrations were observed when comparing antigen-stimulated responses to their group’s respective baseline IFN-γ values. This finding remained consistent across varying antigen concentrations, ranging from 100 µg/mL to 0.1 µg/mL. Stimulation with the full-length KEX1 antigen did not significantly increase IFN-γ from the baseline in any group (Table 4 and Table 5). In contrast, when stimulated with the full-length MSG1 antigen, IFN-γ concentrations were significantly elevated in the healthy control group relative to its baseline values (*p* = 0.0188, Table 4 and Table 5, Figure 2). However, no significant differences in IFN-γ levels were observed between the healthy control group and the group of proven PJP cases. Additionally, in the group of proven PJP cases, stimulation with the full-length MSG1 antigen did not yield significant deviations from the group’s baseline IFN-γ concentrations. Similar results were observed following stimulation with PJ-MIX, whereas the healthy control group demonstrated significantly higher IFN-γ concentrations compared to the group’s baseline value (*p* = 0.0002, Table 4 and Table 5, Figure 3), while the IFN-γ concentrations after stimulation with the PJ-MIX between the two tested cohorts did not differ significantly. Additionally, the response to stimulation with the PJ-MIX did not show significant differences in IFN-γ concentrations in the group of proven PJP cases compared to the group’s baseline value. Table 4, Table 5, Table 6 and Table 7 provide a comprehensive summary of all measured IFN-γ concentrations following in vitro cell stimulation under the respective experimental conditions.

### 3.3. IL-2 Release After Stimulation

**Figure 4:**
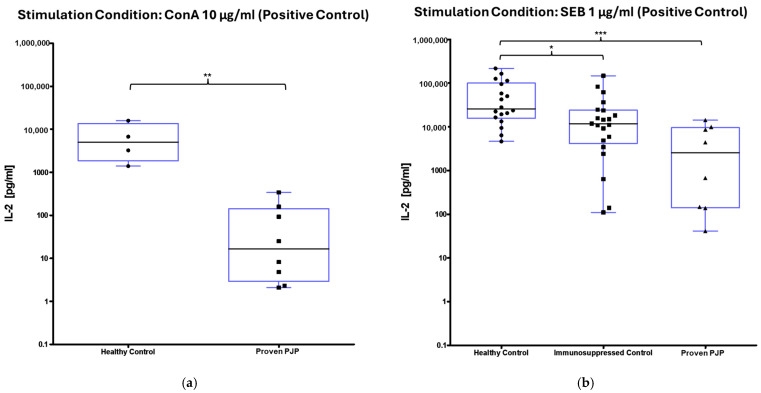
IL-2 concentrations across all cohorts following reference antigen stimulation (positive control). ^1^ (**a**) ConA significantly increased IL-2 in healthy controls compared to proven PJP cases (** *p* = 0.0040) (Mann–Whitney U test); (**b**) SEB significantly increased IL-2 in healthy controls compared to proven PJP cases (*** *p* = 0.0004) and the immunocompromised controls (* *p* = 0.0499) (Kruskal–Wallis test with Dunn’s multiple comparisons). ^1^ Circles, squares and triangles represent the respective measurement of one participant from the cohort specified at the bottom of the figure.

**Figure 5:**
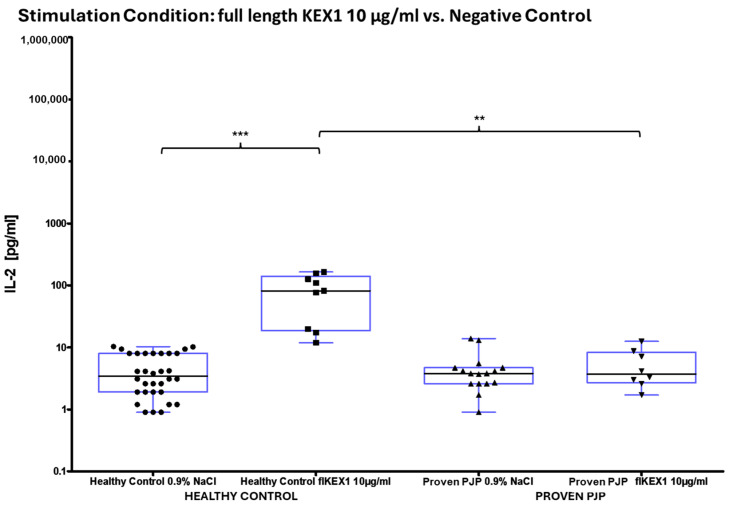
^1^ IL-2 concentrations in healthy controls vs. proven PJP cases following full-length KEX1 stimulation compared to the baseline (NaCl). KEX1 significantly increased IL-2 in healthy controls compared to proven PJP cases (** *p* = 0.0095) and relative to its baseline (*** *p* < 0.0001) (Kruskal–Wallis test with Dunn’s multiple comparisons). ^1^ Circles, squares and triangles represent the respective measurement of one participant from the cohort and stimulation condition specified at the bottom of the figure.

**Figure 6:**
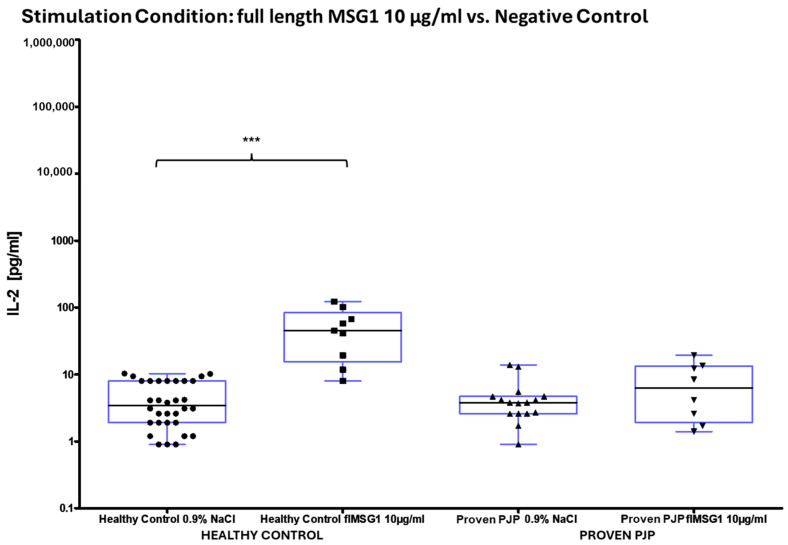
^1^ IL-2 concentrations in healthy controls vs. proven PJP cases After full-length MSG1 stimulation compared to the baseline (NaCl). MSG1 significantly increased IL-2 in healthy controls relative to the baseline (*** *p* < 0.0001) (Kruskal–Wallis test with Dunn’s multiple comparisons). ^1^ Circles, squares and triangles represent the respective measurement of one participant from the cohort and stimulation condition specified at the bottom of the figure.

**Figure 7:**
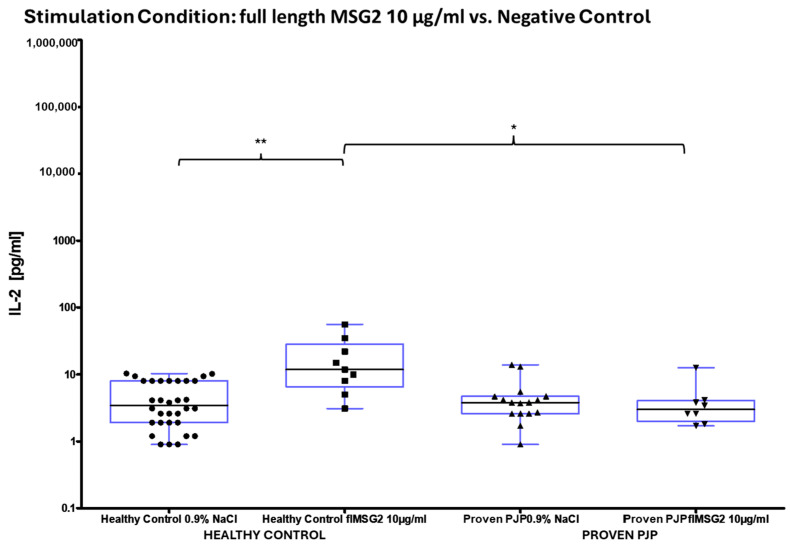
^1^ IL-2 concentrations in healthy controls vs. proven PJP cases following full-length MSG2 stimulation compared to the baseline (NaCl). MSG2 significantly increased IL-2 in healthy controls compared to both proven PJP cases (* *p* = 0.0175) and the baseline (** *p* = 0.0038) (Kruskal–Wallis test with Dunn’s multiple comparisons). ^1^ Circles, squares and triangles represent the respective measurement of one participant from the cohort and stimulation condition specified at the bottom of the figure.

**Figure 8:**
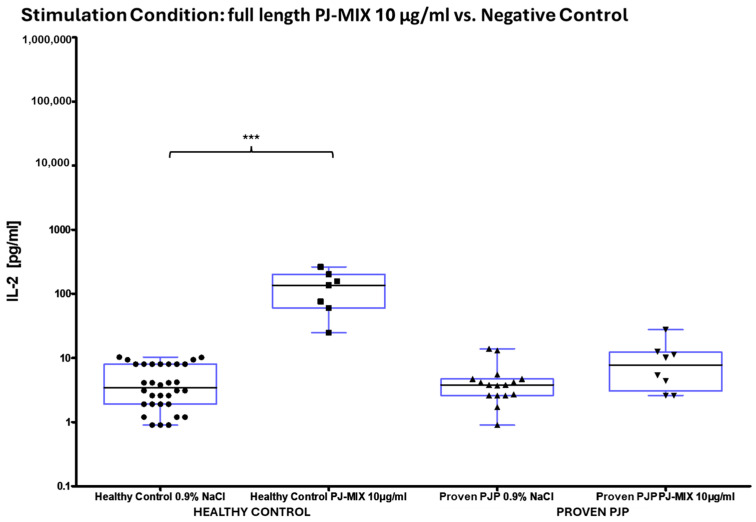
^1^ IL-2 concentrations in healthy controls vs. proven PJP cases following PJ-MIX stimulation compared to the baseline (NaCl). PJ-MIX significantly increased IL-2 in healthy controls relative to the baseline (*** *p* < 0.0001) (Kruskal–Wallis test with Dunn’s multiple comparisons). ^1^ Circles, squares and triangles represent the respective measurement of one participant from the cohort and stimulation condition specified at the bottom of the figure.

The concentration of IL-2 following ConA stimulation was markedly elevated in the healthy control group compared to the group of proven PJP cases (*p* = 0.0040, Table 8 and Figure 4a). Similarly, IL-2 levels after SEB stimulation were significantly higher in the healthy control group than in the group of proven PJP cases (*p* = 0.0004, Table 8 and Figure 4b) and also exceeded those observed in the immunocompromised control group (*p* = 0.0499, Table 8 and Figure 4b). However, IL-2 levels after SEB stimulation were not significantly higher in the immunocompromised control group compared to the group of proven PJP cases. Baseline IL-2 levels, defined as concentrations following stimulation with 0.9% sodium chloride, did not differ across the cohorts. In response to stimulation with the full-length KEX1 antigen, the healthy control group exhibited significantly higher IL-2 concentrations compared to the group of proven PJP cases (*p* = 0.0095, Table 9 and Figure 5). Moreover, IL-2 levels after KEX1 stimulation were substantially elevated relative to the healthy control group’s baseline values (*p* < 0.0001; Table 8 and Table 9, Figure 5). Similarly, full-length MSG1 stimulation resulted in significantly higher IL-2 levels in the healthy control group when compared to the healthy control group’s baseline values (*p* < 0.0001, Table 8 and Table 9 and Figure 6). Consistent findings were observed with full-length MSG2 stimulation, where IL-2 concentrations in the healthy control group significantly surpassed those in the group of proven PJP cases (*p* = 0.0175, Table 9 and Figure 7). Additionally, IL-2 levels following MSG2 stimulation were markedly higher compared to the healthy control group’s baseline levels (*p* = 0.0038, Table 8 and Table 9, Figure 7). Stimulation with PJ-MIX similarly induced significantly greater IL-2 production in the healthy control group compared to the healthy control group’s baseline (*p* < 0.0001, Table 8 and Table 9, Figure 8). In contrast, stimulation with the partial MSG2 and partial KEX1 antigens failed to elicit any significant differences in IL-2 release among the cohorts. Additionally, within each cohort, no significant changes in IL-2 concentrations were detected when antigen-stimulated responses were compared to their respective baseline IL-2 concentration levels. This observation remained consistent across varying antigen concentrations, ranging from 100 µg/mL to 0.1 µg/mL. Table 8, Table 9, Table 10 and Table 11 comprehensively summarize all measured IL-2 concentrations under the experimental conditions described.

### 3.4. IL-17A and IL-17F Release After Stimulation

The measurements of IL-17A and IL-17F concentrations were performed as representative examples. Due to the small numbers of *N* = 4 samples, significance levels were not statistically interpretable. Nonetheless, an analysis of the mean values, medians, and SEM indicated that IL-17A release following stimulation with SEB was consistent across all cohorts. In contrast, IL-17A release after stimulation with ConA was notably lower compared to SEB, and the healthy cohort exhibited a stronger IL-17A response than the other groups (Table 12). Furthermore, IL-17A release in response to PJP antigens remained largely stable in both the healthy and immunocompromised control groups. However, in the group of proven PJP cases, stimulation with, in particular, MSG1 resulted in a two-fold increase in IL-17A release compared to the baseline value within the same group (Table 13). An analysis of the mean values, medians, and SEM indicated that IL-17F release following stimulation with SEB and ConA only diverged from baseline levels in the healthy control group (Table 14). In contrast, IL-17F release after stimulation with *PJ* antigens remained largely consistent across all cohorts, closely resembling the baseline IL-17F levels (Table 15). Table 12, Table 13, Table 14 and Table 15 comprehensively summarize all measured IL-17A and IL-17F concentrations under the experimental conditions described.

## 4. Discussion

PJP is recognized as an AIDS-defining illness in HIV^+^ individuals, leading to a significant overlap between HIV^+^ patients and those suffering from PJP. Since HIV specifically infects CD4^+^ T-cells, it diminishes the very subset of immune cells critical for the immune response evaluated in IGRAs. Overall, the role of IFN-γ in HIV^+^ patients with PJP remains controversial. One study using a mouse model demonstrated that endogenous IFN-γ can promote Pneumocystis immune escape by inhibiting macrophage phagocytosis [49]. These findings are consistent with our observation that PJP patients exhibited elevated baseline IFN-γ levels. Then again, this heightened baseline IFN-γ level in confirmed PJP cases could also be explained by the potential development of immune reconstitution inflammatory syndrome (IRIS) in HIV^+^ individuals following PJP, particularly after initiating antiretroviral therapy [50,51]. The elevated baseline IFN-γ levels complicated the accurate assessment of actual stimulation responses in our experiments. These insights highlight the need to explore whether a CRA, independent of IFN-γ, could be more reliable in the context of HIV^+^ PJP patients. Consequently, we shifted our focus to IL-2 concentrations following stimulation as an alternative approach to mitigate the confounding effects of high baseline IFN-γ levels. Compared to the IFN-γ concentrations after stimulation with the *PJ* antigens, the IL-2 concentrations were elevated relative to the baseline value—two-fold higher at times in both the healthy individuals and those with proven PJP, although not to the same extent as the positive control stimulations with ConA or SEB. Interestingly, the IL-2 concentrations observed after stimulation with all three full-length antigens—KEX1, MSG1, and MSG2—were elevated in healthy individuals compared to those with proven PJP. As a limitation for this study in assessing individual cytokine levels, their multifactorial and complex regulation in vivo must be considered. In this context, our limited patient population poses a risk of bias. Clinically, this finding indicates that a healthy immune system can mount an effective T-cell-mediated defense against *PJ*, whereas the immune response in the individuals with PJP is compromised, contributing to the disease. It is also fascinating that stimulation with the PJ-Mix, containing all three full-length antigens, elicited greater cytokine release than KEX1, MSG1, or MSG2 individually, demonstrating an additive effect in the CRA. This additive phenomenon was observed for both IL-2 and IFN-γ responses. The observation that the titration of partial KEX1 and MSG2 did not increase cytokine release compared to the baseline, while full-length KEX1 and MSG2 did, raises questions about the selection of immunogenic sequences as well as the potential impact of the surface fixation of full-length antigens in our prepared sample tubes versus their soluble partial counterparts. This difference in methodological conditions does not allow for the general conclusion that full-length antigens inherently exhibit higher immunogenicity or induce a stronger immune response. Nonetheless, this finding leads to the hypothesis that the spatial proximity between antigens may also hold immunological importance, potentially influencing the magnitude of the possible immune response. As the cytokine response following stimulation with SEB as a positive control consistently exceeded that observed after stimulation with the lectin ConA across all cohorts, we concluded that SEB represents the optimal antigen for use as a positive control. Due to the poor IL-17A and IL-17F release observed upon stimulation with SEB and ConA in positive controls—particularly in healthy individuals—compared to the robust release of IL-2 and IFN-γ, we did not further investigate these cytokines for our CRA. Immunologically, IL-17 and IFN-γ are produced by distinct subsets of T-helper cells, known as Th17 and Th1-cells, respectively. These cytokines engage in a complex regulatory interplay, where IFN-γ can inhibit Th17 differentiation, while IL-17 may influence Th1 responses under specific conditions [52,53]. Consequently, this aspect could reflect a more differentiated immunological response, emphasizing the intricate connections and regulatory mechanisms between immune pathways. However, among the two, IL-17A showed slightly better performance than IL-17F in the single PJP case we analyzed, particularly following stimulation with the *PJ* antigens. In our study, we observed diminished cytokine responses to the reference antigens used as positive controls, in immunocompromised individuals and those with confirmed PJP, in contrast to the responses observed in healthy individuals. This finding underscores the significant challenges in the clinical application of CRAs in immunocompromised individuals, as commercially developed CRAs often rely on cutoff values calibrated based on healthy, immunocompetent individuals. To address this limitation, future CRA development should prioritize ensuring suitability for patient populations most likely to benefit from the diagnostic and clinical applications of the specific CRA. One potential approach would be to adjust cutoff values according to the immune status of the individual being assessed, incorporating factors such as cytokine release corresponding cell counts and adjusting the cut-off values accordingly. In our study, we found that the T-lymphocyte count, CD4^+^ T-helper-lymphocyte count, and CD4/CD8 ratio were higher in the healthy control group compared to the individuals with proven PJP. Our observation is linked to the co-incidence of PJP and HIV in 6/8 PJP patients from the study resulting in the HIV-typic decrease of this ratio as defined in stage C3 AIDS disease. However, given the limited sample size of proven PJP cases, this group may not be fully representative.

As demonstrated in several studies by Dammermann et al., IFN-γ production can be modulated by various factors. For instance, complement factors C3a and C5a [54], along with other cytokines, influence IFN-γ release and production. In evaluations of IGRA test systems for CMV diagnostics, toll-like receptor 2 agonists, such as lipoteichoic acid (LTA) and peptidoglycans (PGN), were added to the stimulation conditions alongside CMV -specific antigens to enhance IFN-γ production, to improve the sensitivity and thereby the clinical reliability of IGRA test systems. LTA and PGN activate toll-like receptor 2 (TLR-2) and initiate a signaling cascade that boosts cytokine expression in immune cells carrying TLR-2 [36,55]. A similar co-stimulation approach could be considered if cytokine release after stimulation with *PJ* antigens is notably low. Moreover, coupling cytokine release measurement after *PJ* antigen stimulation with additional biomarkers is recommended. For example, Shen et al. observed increased Interleukin-6 (IL-6) release due to Pneumocystis adherence to alveolar epithelium, suggesting that IL-6 could be a valuable additional biomarker. Clinically, β-D-glucan levels are often assessed in peripheral blood when fungal infections are suspected, as *PJ* cysts also incorporate β-D-glucan into their cell walls, making β-D-glucan a relevant additional marker [6,56,57,58]. Lactate dehydrogenase (LDH), an enzyme involved in anaerobic glycolysis and present in the cytoplasm of all cells, is another commonly used clinical biomarker indicating cell death. Some studies have reported elevated serum LDH concentrations in the context of PJP. However, Vogel et al. demonstrated that increased serum LDH was only 43% specific and 63% sensitive for diagnosing PJP [59]. Particularly in the context of cancer patients, LDH is not a suitable biomarker, as it fails to differentiate between elevations due to active cancer-associated cell proliferation and those resulting from a PJP infection. A potential strategy to enhance diagnostic accuracy could involve analytically correlating cytokine release measurements following *PJ* antigen stimulation with the determination of LDH and β-D-glucan levels. Even though our current approach to PJP diagnostics using indirect CRA-based test methods may not yet meet modern laboratory diagnostic standards in terms of sensitivity and specificity, these methods could still provide considerable added value when used in combination, as they are easily accessible and resource-efficient.

To generate robust insights, a balanced distribution of cohorts in terms of age and gender is essential. Since more men were included in this study, questions arise regarding the comparability of sex-specific immunological responses. Although the results did not reveal significant differences between male and female subjects, this unequal distribution of sexes may still be considered as a potential confounder. Results from other studies also suggest geographic variations in *PJ* [24], which were not addressed in our study. The recruitment phase for this multi-center study conducted at four hospitals in Berlin and Brandenburg in Germany lasted 12 months, during which only a relatively small number of confirmed PJP cases (*N* = 8) were identified. This may reflect the effectiveness of established clinical practices for prophylaxis in patients with a predictably high risk of PJP, such as those undergoing oncology treatments or prolonged steroid therapy. Additionally. this could also be attributed to a significant under-awareness of considering PJP as a differential diagnosis in cases with corresponding clinical symptoms.

A potential future clinical application of our findings on *PJ* antigens and CRAs lies in their use for monitoring effective endogenous T-cell-mediated defenses against PJP. It is important to acknowledge that our pilot study provides a preliminary basis for further exploration of these aspects in larger, more comprehensive studies. Our peripheral T-cell-based CRA could substantially streamline the diagnosis of PJP by providing a less invasive alternative to BAL with PCR, the current gold standard. Because BAL requires specialized equipment, trained personnel, and involves procedural risks—particularly for critically ill or immunocompromised patients—it is often impractical in resource-limited settings. In contrast, obtaining peripheral blood is simpler, safer, and easier to perform, significantly expanding diagnostic capacity. Beyond reducing procedural risks, our PJP-specific CRA could enable earlier detection and screening of high-risk groups—such as immunocompromised individuals and/or those on immunosuppressive therapy, pediatric patients or HIV^+^ individuals with borderline but not yet critically low CD4^+^ cell counts, or those on immunosuppressive therapy—by identifying those lacking a probably protective T-cell immune response to *PJ*. This allows for timely prophylaxis or more frequent monitoring. The test may also guide therapy decisions in patients presenting with respiratory symptoms and diffuse CT detected lung infiltrates when the etiology is unclear. Furthermore, implementation could be both cost-effective and logistically feasible; in a commercial setting with widespread application, the assay would be conducted in test tubes pre-coated with the relevant antigens, requiring only the pipetting of peripheral blood before proceeding with an analysis modeled similar to clinically used IGRA systems. Although further validation is necessary to confirm its sensitivity and specificity, our peripheral PJP-specific CRA holds great promise for safer, more accessible, and potentially more efficient diagnosis and management of PJP—especially in resource-limited regions—and could ultimately reduce morbidity and mortality through earlier prophylaxis administration. Further studies shall address this indication in a larger setting.

## 5. Conclusions

This pilot study highlights the potential of assessing IL-2 and IFN-γ release following stimulation with full-length *PJ* antigens, including KEX1, MSG1, and the PJ-Mix antigen combination, as a tool for evaluating immune responses. Full-length MSG2 also showed promise for IL-2 release. Notably, PJ-Mix stimulation elicited the strongest cytokine responses in both IL-2 and IFN-γ, indicating a cumulative and synergistic effect that warrants further investigation. The consistently robust cytokine response observed with SEB as a positive control across all cohorts underscores its suitability as the optimal antigen for this purpose, outperforming the lectin ConA. In HIV^+^ PJP patients, elevated baseline IFN-γ levels complicated the accurate assessment of stimulation responses. In contrast, IL-2 emerged as the more suitable cytokine for a PJP-specific CRA, including the HIV^+^ individuals. Future studies should validate these findings by exploring higher titration levels of full-length antigens, integrating co-stimulatory agents to improve the detection of weak cellular responses currently below measurable thresholds, and additionally incorporating complementary biomarkers such as IL-6, LDH, and β-D-glucans. In summary, this pilot study establishes a foundation for developing a PJP-specific CRA, with the significant potential to advance PJP diagnostics and deepen our understanding of T-cell immunity against *PJ*. In a broader context, our project could contribute to the development of a resource-efficient diagnostic approach to bridge a critical gap in the detection of PJP or, at the very least, help identify individuals at high risk of contracting PJP. This is particularly relevant in regions with limited healthcare resources, incomplete access to HIV therapy, and consequently, a higher risk for PJP. Moreover, such a diagnostic method could be especially valuable for patients in whom invasive bronchoscopy and BAL for direct pathogen detection are not feasible due to their vulnerable and compromised health status.

## Figures and Tables

**Table 1:** Characteristics of all participants in this study.

Characteristics				
		Healthy Individuals	Immunocompromised Individuals ^1^	Individuals with Proven PJP
Total *N* = 50		22 (44%)	20 (40%)	8 (16%)
	Male	12 (55%)	12 (60%)	8 (100%)
	Female	10 (45%)	8 (40%)	-
Age (Years) ^2^		41 ± 17	66 ± 12	46 ± 23

^1^ Excluding the PJP cases. ^2^ The age is presented as mean ± S.D.

**Table 2:** The stimulation conditions.

Condition Number	Stimulation Condition	Composition	Final Antigen Concentration in Blood [µg/mL]
1	Negative control (Baseline): 0.9% sodium chloride (NaCl)	500 µL whole blood +50 µL 0.9% NaCl solution +5 µL 1 mg/mL glucose solution	-
2	Positive control: SEB	500 µL whole blood +50 µL SEB 1 µg/mL +5 µL 1 mg/mL glucose solution	1
3	Positive control: Concanavalin A	500 µL whole blood	10
4	KEX1 partial (aa 291–380) 100 µg/mL	500 µL whole blood +50 µL KEX1 partial +5 µL 1 mg/mL glucose solution	100
5	KEX1 partial (aa 291–380) 50 µg/mL	500 µL whole blood +50 µL KEX1 partial +5 µL 1 mg/mL glucose solution	50
6	KEX1 partial (aa 291–380) 10 µg/mL	500 µL whole blood +50 µL KEX1 partial +5 µL 1 mg/mL glucose solution	10
7	KEX1 partial (aa 291–380) 5 µg/mL	500 µL whole blood +50 µL KEX1 partial +5 µL 1 mg/mL glucose solution	5
8	KEX1 partial (aa 291–380) 1 µg/mL	500 µL whole blood +50 µL KEX1 partial +5 µL 1 mg/mL glucose solution	1
9	KEX1 partial (aa 291–380) 0.5 µg/mL	500 µL whole blood +50 µL KEX1 partial +5 µL 1 mg/mL glucose solution	0.5
10	KEX1 partial (aa 291–380) 0.1 µg/mL	500 µL whole blood +50 µL KEX1 partial +5 µL 1 mg/mL glucose solution	0.1
11	MSG2 partial (aa 32–72, 170–208, 264–305) 100 µg/mL	500 µL whole blood +50 µL MSG2 partial +5 µL 1 mg/mL glucose solution	100
12	MSG2 partial (aa 32–72, 170–208, 264–305) 50 µg/mL	500 µL whole blood +50 µL MSG2 partial +5 µL 1 mg/mL glucose solution	50
13	MSG2 partial (aa 32–72, 170–208, 264–305) 10 µg/mL	500 µL whole blood +50 µL MSG2 partial +5 µL 1 mg/mL glucose solution	10
14	MSG2 partial (aa 32–72, 170–208, 264–305) 5 µg/mL	500 µL whole blood +50 µL MSG2 partial +5 µL 1 mg/mL glucose solution	5
15	MSG2 partial (aa 32–72, 170–208,264–305) 1 µg/mL	500 µL whole blood +50 µL MSG2 partial +5 µL 1 mg/mL glucose solution	1
16	MSG2 partial (aa 32–72, 170–208, 264–305) 0.5 µg/mL	500 µL whole blood +50 µL MSG2 partial +5 µL 1 mg/mL glucose solution	0.5
17	MSG2 partial (aa 32–72, 170–208, 264–305) 0.1 µg/mL	500 µL whole blood +50 µL MSG2 partial +5 µL 1 mg/mL glucose solution	0.1
18	KEX1 full length 10 µg/mL (EUROIMMUN AG)	500 µL whole blood	10
19	MSG 1 full length 10 µg/mL (EUROIMMUN AG)	500 µL whole blood	10
20	MSG 2 full length 10 µg/mL (EUROIMMUN AG)	500 µL whole blood	10
21	PJ-Mix full length 10 µg/mL (EUROIMMUN AG)	500 µL whole blood	10

**Table 3:** Cell counts of leucocytes and subsets, on the day of cell stimulation. Presented as mean ± SEM with median shown in parentheses.

		Leucocytes	T-Lymphocytes		
	*N*	Leucocytes (CD45^+^) [10^9^/L]	T-Lymphocytes (CD45^+^, CD3^+^) [Cells/µL]	Th-Lymphocytes (CD4^+^) [Cells/µL]	CD4^+^/CD8^+^–Ratio
All	25	7 ± 1 (6)	1109 ± 151 (1087)	553 ± 122 (549)	2 ± 0 (2)
Healthy control	10	7 ± 0 (7)	1524 ± 176 (1391)	938 ± 139 (799)	2 ± 0 (2)
Immunocompromised control	7	10 ± 2 (9)	N/A ^1^	N/A ^1^	N/A ^1^
Proven PJP	8	5 ± 1 (4)	701 ± 140 (800)	118 ± 58 (57)	0 ± 0 (0)

^1^ The immunocompromised control group was not tested under these conditions due to limitations in available biomaterial volumes.

**Table 4:** Total IFN-γ concentrations after stimulation with reference antigens. Presented as mean ± SEM and median shown in parentheses.

IFN-γ [pg/mL]		Negative Control	Positive Controls	
	*N*	NaCl	SEB	ConA ^1^
All	50	12 ± 4 (3)	25,337 ± 4896 (8995)	2583 ± 800 (670)
Healthy control	22	3 ± 1 (3)	47,760 ± 9046 (32,220)	4683 ± 1247 (6104)
Immunocompromised control	20	9 ± 3 (3)	9470 ± 3033 (1277)	N/A ^2^
Proven PJP	8	54 ± 15 (27)	5508 ± 2084 (3408)	528 ± 292 (145)

^1^ The immunocompromised control group was not tested under these conditions due to limitations in available biomaterial volumes. ^2^ Due to limitations in the available biomaterial volumes, only the proven PJP cases and an equal number of age- and gender-matched healthy controls were tested under these conditions.

**Table 5:** Total IFN-γ concentration after stimulation with full-length *PJ* antigens KEX1, MSG1, MSG2, and the PJ-MIX in a predetermined concentration of 10 µg/mL. Presented as mean ± SEM and median shown in parentheses.

IFN-γ [pg/mL]	*N*	Full Length KEX1 10 µg/mL	Full Length MSG1 10 µg/mL	Full Length MSG2 10 µg/mL	Full Length PJ-MIX 10 µg/mL
All	17 ^1^	42 ± 12 (20)	52 ± 18 (17)	34 ± 11 (16)	67 ± 19 (23)
Healthy control	9	31 ± 14 (15)	52 ± 30 (14)	17 ± 9 (3)	82 ± 32 (26)
Proven PJP	8	60 ± 22 (33)	59 ± 22 (32)	58 ± 20 (40)	59 ± 23 (27)

^1^ Due to limitations in the available biomaterial volumes, only the proven PJP cases and an equal number of age- and gender-matched healthy controls were tested under these conditions.

**Table 6:** Total IFN-γ concentration after stimulation with partial KEX1 in different concentrations. Presented as mean ± SEM and median shown in parentheses.

IFN-γ [pg/mL]								
Partial KEX1	*N*	100 µg/mL	50 µg/mL	10 µg/mL	5 µg/mL	1 µg/mL	0.5 µg/mL	0.1 µg/mL
All	35	17 ± 7 (3)	17 ± 7 (3)	13 ± 4 (3)	17 ± 7 (3)	15 ± 6 (3)	16 ± 7 (3)	15 ± 6 (3)
Healthy control	14	3 ± 1 (3)	3 ± 1 (3)	3 ± 1 (3)	2 ± 0 (3)	2 ± 1 (3)	3 ± 1 (3)	2 ± 0 (3)
Immunocompromised control	13	6 ± 3 (3)	6 ± 3 (3)	9 ± 4 (3)	6 ± 3 (3)	7 ± 3 (3)	6 ± 3 (3)	6 ± 3 (3)
Proven PJP	8	53 ± 23 (24)	53 ± 22 (26)	52 ± 22 (24)	54 ± 23 (29)	47 ± 20 (30)	52 ± 21 (32)	48 ± 20 (24)

**Table 7:** Total IFN-γ concentration after stimulation with partial MSG2 in different concentrations. Presented as mean ± SEM and median shown in parentheses.

IFN-γ [pg/mL]								
Partial MSG2	*N*	100 µg/mL	50 µg/mL	10µg/mL	5 µg/mL	1 µg/mL	0.5 µg/mL	0.1 µg/mL
All	30	23 ± 9 (5)	23 ± 8 (6)	19 ± 6 (4)	21 ± 8 (6)	23 ± 8 (7)	21 ± 7 (6)	23 ± 8 (4)
Healthy control	9	N/A ^1^	N/A ^1^	4 ± 1 (3)	N/A ^1^	N/A ^1^	N/A ^1^	N/A ^1^
Immunocompromised control	13	11 ± 4 (4)	12 ± 5 (5)	11 ± 4 (4)	10 ± 4 (5)	11 ± 4 (6)	11 ± 5 (3)	11 ± 5 (3)
Proven PJP	8	53 ± 23 (24)	52 ± 22 (27)	49 ± 20 (24)	48 ± 20 (24)	51 ± 22 (24)	46 ± 18 (24)	53 ± 22 (26)

^1^ The healthy control group was not tested under these conditions due to limitations in available biomaterial volumes.

**Table 8:** Total IL-2 concentration after stimulation with reference antigens. Presented as mean ± SEM and median shown in parentheses.

IL-2 [pg/mL]		Negative Control	Positive Controls	
	*N*	NaCl	SEB	ConA ^1^
All	47	4 ± 1 (3)	36,404 ± 7046 (15,291)	2146 ± 1266 (93)
Healthy Control	18	5 ± 1	57,300 ± 14,420 (25,910)	6815 ± 3212 (5.007)
Immunocompromised Control	21	4 ± 1	23,860 ± 7645 (11,800)	N/A ^2^
Proven PJP	8	5 ± 1	4766 ± 1947 (2542)	79 ± 42 (17)

^1^ Due to limitations in the available biomaterial volumes, only the proven PJP cases and an equal number of age- and gender-matched healthy controls were tested under these conditions. ^2^ The immunocompromised control group was not tested under these conditions due to limitations in available biomaterial volumes.

**Table 9:** Total IL-2 concentration after stimulation with full-length KEX1, MSG1, MSG2, and the PJ-MIX in a predetermined concentration of 10 µg/mL. Presented as mean ± SEM and median shown in parentheses.

IL-2 [pg/mL]	*N*	Full Length KEX1 10 µg/mL	Full Length MSG1 10 µg/mL	Full Length MSG2 10 µg/mL	Full Length PJ-MIX 10 µg/mL
All	17 ^1^	45 ± 14 (12)	31 ± 9 (14)	11 ± 3 (5)	63 ± 21 (19)
Healthy Control	9	85 ± 20 (82)	58 ± 14 (52)	18 ± 6 (12)	131 ± 32 (136)
Proven PJP	8	5 ± 1 (4)	8 ± 2 (6)	4 ± 1 (3)	10 ± 3 (8)

^1^ The immunocompromised control group was not tested under these conditions due to limitations in available biomaterial volumes.

**Table 10:** Total IL-2 concentration after stimulation with partial KEX1 in different concentrations. Presented as mean ± SEM and median shown in parentheses.

IL-2 [pg/mL]								
Partial KEX1	*N*	100 µg/mL	50 µg/mL	10 µg/mL	5 µg/mL	1 µg/mL	0.5 µg/mL	0.1 µg/mL
All	35	4 ± 1 (3)	5 ± 1 (3)	4 ± 1 (3)	4 ± 1 (3)	4 ± 1 (3)	6 ± 3 (3)	4 ± 1 (2)
Healthy Control	14	3 ± 1 (2)	4 ± 2 (2)	4 ± 2 (3)	2 ± 0 (2)	2 ± 0 (2)	7 ± 5 (2)	2 ± 0 (2)
Immunocompromised Control	13	6 ± 2 (2)	6 ± 2 (4)	4 ± 1 (2)	6 ± 2 (2)	6 ±2 (3)	6 ± 2 (2)	5 ± 2 (2)
Proven PJP	8	4 ± 1 (4)	4 ± 1 (4)	5 ± 1 (3)	4 ± 1 (4)	5 ± 1 (4)	5 ± 1 (3)	5 ± 1 (4)

**Table 11:** Total IL-2 concentration after stimulation with partial MSG2 in different concentrations. Presented as mean ± SEM and median shown in parentheses.

IL-2 [pg/mL]								
Partial MSG2	*N*	100 µg/mL	50 µg/mL	10 µg/mL	5 µg/mL	1 µg/mL	0.5 µg/mL	0.1 µg/mL
All	30	3 ± 0 (3)	3 ± 1 (3)	5 ± 1 (3)	3 ± 1 (3)	3 ± 1 (3)	3 ± 1 (3)	3 ± 1 (3)
Healthy Control	9	N/A ^1^	N/A ^1^	8 ± 2 (8)	N/A ^1^	N/A ^1^	N/A ^1^	N/A ^1^
Immunocompromised Control	13	4 ± 1 (2)	4 ± 1 (2)	3 ± 1 (2)	3 ± 1 (2)	3 ± 1 (2)	3 ± 1 (2)	3 ± 1 (2)
Proven PJP	8	4 ± 1 (4)	4 ± 1 (4)	5 ± 1 (4)	4 ± 1 (4)	4 ± 1 (4)	5 ± 1 (4)	5 ± 1 (4)

^1^ The healthy control group was not tested under these conditions due to limitations in available biomaterial volumes.

**Table 12:** Total IL-17A concentration after stimulation with reference antigens and partial KEX1 and MSG2. Presented as mean ± SEM and median shown in parentheses.

IL-17A [pg/mL]		Negative Control	Positive Controls		Partial Antigens	
	*N*	NaCl ^1^	SEB	ConA	Partial KEX1 10 µg/mL	Partial MSG2 10 µg/mL
All	4 ^1^	4 ± 2 (3)	253 ± 0 (253)	46 ± 17 (31)	3 ± 0 (3)	5 ± 2 (3)
Healthy Control	2	3 ± 0 (3)	253 ± 0 (253)	67 ± 30 (67)	3 ± 0 (3)	3 ± 0 (3)
Immunocompromised Control	1	3 ± 0 (3)	253 ± 0 (253)	25 ± 0 (25)	3 ± 0 (3)	3 ± 0 (3)
Proven PJP	1	6 ± 2 (6)	253 ± 0 (253)	25 ± 0 (25)	3 ± 0 (3)	11 ± 0 (11)

^1^ Due to limited biomaterial availability, a small representative subset from each cohort was randomly selected for sample measurement.

**Table 13:** Total IL-17A concentration after stimulation with full-length KEX1, MSG1, MSG2, and the PJ-MIX in a predetermined concentration of 10 µg/mL. Presented as mean ± SEM and median shown in parentheses.

IL-17A [pg/mL]	*N*	Full Length KEX1 10 µg/mL	Full Length MSG1 10 µg/mL	Full Length MSG2 10 µg/mL	Full Length PJ-MIX 10 µg/mL
All	4 ^1^	4 ± 2 (3)	5 ± 3 (3)	3 ± 1 (3)	3 ± 1 (3)
Healthy Control	2	3 ± 0 (3)	2 ± 0 (2)	3 ± 0 (3)	3 ± 0 (3)
Immunocompromised Control	1	3 ± 0 (3)	2 ± 0 (2)	3 ± 0 (3)	3 ± 0 (3)
Proven PJP	1	10 ± 0 (10)	13 ± 0 (13)	6 ± 0 (6)	5 ± 0 (5)

^1^ Due to limited biomaterial availability, a small representative subset from each cohort was randomly selected for sample measurement.

**Table 14:** Total IL-17F concentration after stimulation with reference antigens and partial KEX1 and MSG2. Presented as mean ± SEM and median shown in parentheses.

IL-17F [pg/mL]		Negative Control	Positive Controls		Partial Antigenes	
	*N*	NaCl	SEB	ConA	Partial KEX1 10 µg/mL	Partial MSG2 10 µg/mL
All	4 ^1^	3 ± 0 (3)	6 ± 2 (5)	13 ± 10 (5)	3 ± 0 (3)	3 ± 0 (3)
Healthy Control	2	3 ± 0 (3)	9 ± 4 (9)	33 ± 0 (33)	3 ± 0 (3)	3 ± 0 (3)
Immunocompromised Control	1	3 ± 0 (3)	5 ± 0 (5)	5 ± 0 (5)	3 ± 0 (3)	3 ± 0 (3)
Proven PJP	2	3 ± 0 (3)	4 ± 1 (4)	3 ± 0 (3)	3 ± 0 (3)	3 ± 0 (3)

^1^ Due to limited biomaterial availability, a small representative subset from each cohort was randomly selected for sample measurement.

**Table 15:** Total IL-17F concentration after stimulation with full-length KEX1, MSG1, MSG2, and the PJ-MIX in a predetermined concentration of 10 µg/mL. Presented as mean ± SEM and median shown in parentheses.

IL-17F [pg/mL]	*N*	Full Length KEX1 10 µg/mL	Full Length MSG1 10 µg/mL	Full Length MSG2 10 µg/mL	Full Length PJ-MIX 10 µg/mL
All	3 ^1^	3 ± 0 (3)	3 ± 0 (3)	3 ± 0 (3)	3 ± 0 (3)
Healthy Control	1	3 ± 0 (3)	3 ± 0 (3)	3 ± 0 (3)	3 ± 0 (3)
Immunocompromised Control	1	3 ± 0 (3)	3 ± 0 (3)	3 ± 0 (3)	3 ± 0 (3)
Proven PJP	1	3 ± 0 (3)	3 ± 0 (3)	3 ± 0 (3)	3 ± 0 (3)

^1^ Due to limited biomaterial availability, a small representative subset from each cohort was randomly selected for sample measurement.

## Data Availability

The original contributions presented in this study are included in the article. Further inquiries can be directed to the corresponding author.
